# Systematic Review: Allogenic Use of Stromal Vascular Fraction (SVF) and Decellularized Extracellular Matrices (ECM) as Advanced Therapy Medicinal Products (ATMP) in Tissue Regeneration

**DOI:** 10.3390/ijms21144982

**Published:** 2020-07-15

**Authors:** Pietro Gentile, Aris Sterodimas, Jacopo Pizzicannella, Laura Dionisi, Domenico De Fazio, Claudio Calabrese, Simone Garcovich

**Affiliations:** 1 Department of Surgical Science, Plastic and Reconstructive Surgery, “Tor Vergata” University, 00133 Rome, Italy; 2 Scientific Director of AIRMESS, Academy of International Regenerative Medicine & Surgery Societies, 1201 Geneva, Switzerland; 3 Department of Plastic and Reconstructive Surgery, Metropolitan General Hospital, 18547 Athens, Greece; aris@sterodimas.com; 4 ASL02 Lanciano-Vasto Chieti, Ss. Annunziata Hospital, 66100 Chieti, Italy; jacopo.pizzicannella@unich.it; 5 Dionisi Law Firm, 00133 Rome, Italy; studio@avvocatolauradionisi.it; 6 Institute of Plastic Surgery, Galeazzi Hospital, 20122 Milan, Italy; info@domenicodefazio.it; 7 San Rossore Breast Unit, 56122 Pisa, Italy; claudiocalabrese.it@gmail.com; 8 Institute of Dermatology, F. Policlinico Gemelli IRCSS, Università Cattolica del Sacro Cuore, 00168 Rome, Italy; simgarko@yahoo.it

**Keywords:** allogenic SVF, allogenic fat graft, allogenic ASCs, allogenic fat transplants, fat graft, adipose stem cells, ASCs, stromal vascular fraction, SVF

## Abstract

Stromal vascular fraction (SVF) containing adipose stem cells (ASCs) has been used for many years in regenerative plastic surgery for autologous applications, without any focus on their potential allogenic role. Allogenic SVF transplants could be based on the possibility to use decellularized extracellular matrix (ECM) as a scaffold from a donor then re-cellularized by ASCs of the recipient, in order to develop the advanced therapy medicinal products (ATMP) in fully personalized clinical approaches. A systematic review of this field has been realized in accordance with the Preferred Reporting for Items for Systematic Reviews and Meta-Analyses-Protocols (PRISMA-P) guidelines. Multistep research of the PubMed, Embase, MEDLINE, Pre-MEDLINE, PsycINFO, CINAHL, Clinicaltrials.gov, Scopus database, and Cochrane databases has been conducted to identify articles and investigations on human allogenic ASCs transplant for clinical use. Of the 341 articles identified, 313 were initially assessed for eligibility on the basis of the abstract. Of these, only 29 met all the predetermined criteria for inclusion according to the PICOS (patients, intervention, comparator, outcomes, and study design) approach, and 19 have been included in quantitative synthesis (meta-analysis). Ninety-one percent of the studies previously screened (284 papers) were focused on the in vitro results and pre-clinical experiments. The allogenic use regarded the treatment of perianal fistulas, diabetic foot ulcers, knee osteoarthritis, acute respiratory distress syndrome, refractory rheumatoid arthritis, pediatrics disease, fecal incontinence, ischemic heart disease, autoimmune encephalomyelitis, lateral epicondylitis, and soft tissue defects. The information analyzed suggested the safety and efficacy of allogenic ASCs and ECM transplants without major side effects.

## 1. Introduction

The Stromal Vascular Fraction Cells (SVFs) and Adipose Stem Cells (ASCs), both contained in the Stromal Vascular fraction (SVF) portion, meet the majority of the International Society for Cellular Therapy—ISCT’s criteria for Mesenchymal Stem Cells (MSCs) [1].

The ISCT [1] suggested four parameters to define MSCs:

1. MSCs are disc-adherent in standard cultures;
2. MSCs differentiate in adipocytes, chondroblasts, and osteoblasts;
3. MSCs express CD73, CD90, and CD105;
4. MSCs do not express CD11b, CD14, CD19, CD34, CD45, CD79, c-kit, and human leukocyte antigen-DR.

The SVF offers a rich source of ASCs that may be easily gathered from human adipose tissue (HAT) [2]. Generally, each mL of HAT offers 300,000 SVFs, of which 1–3% are represented by ASCs (3000–9000/mL) [3]. Therefore, the SVFs and related ASCs are used for many years in regenerative plastic surgery for autologous implication, without any focus on their potential allogenic role. Additionally, the anti-inflammatory and immunomodulatory activities of SVFs and ASCs make them also considered as a potential cellular therapy in Coronavirus disease 2019 (COVID-19), as preliminarily displayed [4].

These effects have been in vivo displayed by the use of MSCs, via the increased peripheral lymphocytes amount, the decline in the C-reactive protein, and waning of over-activated cytokine-secreting immune cells (CXCR3 + CD4 + T cells, CXCR3 + CD8 + T cells, and CXCR3 + Natural Killer (NK) cells) into the blood of COVID-19 patients, by mean 4.5 days later the MSCs with intravenous administration [5].

As known, the most investigated MSCs are those present in the umbilical cord (UC), bone marrow (BM), and in adipose tissue (AD) called, respectively, UC-MSCs, BM-MSCs, and AD-MSCs [6].

During the last few years, SVF-based autologous cellular therapies have been tested in several clinical settings, like scars [2,7], hemifacial atrophy [8], breast reconstruction [9], wound healing and cancer therapy [10], hair regrowth [11], breast augmentation [12], and in vitro applications [13].

Currently, human tissue-engineering is the science useful in creating a biological scaffold represented by the decellularized extracellular matrix (ECM) [14,15,16] from SVF of a donor that could be re-cellularized with the ASCs of the recipient. The decellularization process permits researchers to obtain cell-free natural ECMs, characterized by an adequate three-dimensional organization and proper molecular composition [17]. To date, allogenic cells and tissues and have been used in different hosts, producing different long-term results. One of the problems identified has been the availability of donor tissues, which often appears limited.

The great fat availability in the human population solves this problem.

HAT may be obtained from gentle and non-invasive liposuction. Banking storage of HAT via the implementation of fat donation could be helpful in the development of new strategies to regenerate damaged tissues. The aim could be to create a Human Fat Bio-Bank, in which the following three stages could be performed:

1. Fat tissue could be obtained by selected donors, checked, and stored;
2. On-demand, fat tissue, and in particular his SVF portion, could be used for autologous or allogenic use. In the last case, fat tissue should be undergone to decellularize so as to obtain a scaffold represented by the decellularized ECM;
3. Decellularized ECM should be re-cellularized with the ASCs of the recipient.

This Human Fat Tissue Bio-Bank could be a valuable source of allogenic tissue for new graft developments.

This systematic review highlighted the importance of a readily available source of HAT to develop ECMs for tissue engineering approaches, detailing the different clinical applications of allogenic ASCs, to date used, and in vivo implantation. Each step of processing tissues should guarantee both the exclusion of disease and infection transmission, in which the strict decellularization, aimed at antigenicity elimination and immunoreactions avoidance safeguard the biomechanical integrity of ECM.

## 2. Results

### 2.1. Description of Included Studies

Of the 341 studies returned from the initial search, 313 were initially assessed for eligibility on the basis of the abstract. Of these, only 29 met all the predetermined criteria for inclusion, and 19 were included in quantitative synthesis (meta-analysis). Ninety-one percent of the studies previously screened (284 papers) were focused on the “in vitro” results and animal experiments. No side effects were reported in the analysis. The human clinical allogenic use regarded the treatment of complex perianal fistulas, diabetic foot ulcers, knee osteoarthritis, acute respiratory distress syndrome (ARDS), refractory rheumatoid arthritis, pediatrics disease, fecal incontinence, allogenic flap, ischemic heart disease, autoimmune encephalomyelitis, lateral epicondylitis, and soft tissue defects.

### 2.2. Allogenic Human Clinical Use of Adipose Stem Cells (ASCs)

Relating to the allogenic human clinical use of ASCs, only 29 studies met all the predetermined criteria for inclusion, and 19 were included in quantitative synthesis (meta-analysis), as aforementioned. Zhao et al. [18] reported the cartilage repair in a randomized clinical trial performed in eighteen patients using allogenic human adipose-derived mesenchymal progenitor cells (haMPCs) in knee osteoarthritis.

In the same field, Kuah D et al. [19] reported in a single-center, randomized, double-blind, placebo-controlled, single ascending dose study, performed on 20 patients affected by symptomatic knee osteoarthritis, the safety, tolerability, and efficacy, obtained by single intra-articular injection of in vitro expanded MSCs derived from donor of HAT.

Álvaro-Gracia et al. [20] evaluated the safety and tolerability of the intravenous infusion of Cx611, a suspension of allogenic expanded ASCs (e-ASCs), in 53 patients with refractory rheumatoid arthritis (RA) through a multicenter, dose-escalation, randomized, blinded, placebo-controlled, phase Ib/IIa clinical trial. The administration of Cx611 was well tolerated, in absence of dose-related toxicity. Additionally, a trend for clinical efficacy wasobserved.

Manferdini et al. [21] examined the impact of different sources of GMP clinical-grade AD-MSCs on inflammatory factors in osteoarthritic (OA) chondrocytes and synoviocytes. The anti-inflammatory impact of AD-MSCs are probably independent of AD-MSC adipose tissue sources and donors but, rather, are dependent on the inflammatory status of OA chondrocytes and synoviocytes. AD-MSCs seem to be able to sense and respond to the local environment, suggesting that AD-MSCs can be useful in osteoarticular diseases treatment.

Dige et al. [22] showed the healing of perianal fistulas in 21 patients who underwent infiltrations of cultured autologous and allogenic ASCs. Six months after the last injection, twelve patients (57%) had complete fistula healing, three patients (14%) had ceased fistula secretion, and one patient (5%) reported reduced secretion. In the same field, Panés et al. [23,24] reported the use of allogenic expanded ASCs (Cx601) in patients with Crohn’s disease and perianal fistulas, concluding that this therapy was safe and effective in closing external openings, compared with placebo.

In addition, Park et al. [25] confirmed the safety and feasibility of allogenic ASCs for the perianal fistula treatment in Crohn’s disease.

De la Portilla et al. [26] confirmed further the safety and efficacy of a suspension of expanded allogenic adipose-derived mesenchymal stem cells (e-a-MSCs) for the perianal complex fistula treatment in patients suffering Crohn’s disease.

Kastrup et al. [27] conducted the first clinical trial in humans evaluating the safety and feasibility of a newly developed and cryopreserved Cardiology Stem Cell Centre adipose-derived stromal cell (CSCC_ASC) product from healthy donors for intramyocardial infiltration in ten patients suffering ischemic heart disease and ischemic heart failure (IHF). They reported an improvement in cardiac function after CSCC_ASC treatment at a 6-month follow-up: left ventricular end-systolic volume was decreased, and the left ventricular ejection fraction was increased. In addition, exercise capacity was increased. The outcomes obtained confirmed that cryopreserved product CSCC_ASC from healthy donors was a safe and feasible therapy.

Jurado et al. [28] evaluated the safety and feasibility of the use of AD-MSCs in patients with chronic graft-versus-host disease (GvHD). Fourteen patients suffering moderate (*n* = 7) or severe (*n* = 7) chronic GvHD received 1 × 10^6^/kg (Group A, *n* = 9) or 3 × 10^6^/kg (Group B, *n* = 5) AD-MSCs with cyclosporine and prednisone as first-line therapy. Ten of the fourteen patients were able to continue under the protocol: 80% were in complete remission, and 100% were off of steroids at week 56. The remaining four patients either worsened from chronic GvHD (*n* = 3) or abandoned the trial (*n* = 1). At the end of the investigation, eleven of fourteen patients were alive (overall survival 71.4%, a median survival of 45.3 weeks). They concluded that AD-MSCs, in combination with immunosuppressive therapy, may be considered feasible and safe and likely would have an impact on the course of chronic GvHD.

Tsai et al. [29] reported the outcomes of phase I/IIa clinical trial to primarily evaluate the safety, tolerability, and, secondarily, the possible efficacy of intravenous infusion of allogenic AD-MSCs from healthy donors in six patients suffering spinocerebellar ataxia type 3 and in one suffering multiple system atrophy-cerebellar types performing intravenous infusion of 10^6^ cells/kg body weight. They concluded that allogenic AD-MSCs is given by intravenous administration appears to be safe and tolerable in patients with spinocerebellar ataxia type 3, thus supporting the advancement of the clinical development of allogenic MSCs for the treatment of spinocerebellar ataxias.

Díez-Tejedor et al. [30] reported a Phase IIa, prospective, randomized, double-blind, placebo-controlled, single-center, pilot clinical trial involving twenty patients showing acute ischemic stroke treated with intravenous infusion of allogenic AD-MSCs or placebo administered as a single intravenous dose within the first 2 weeks after the onset of stroke symptoms. They described the safety and efficacy of intravenous infusion of allogenic AD-MSCs within the first 2 weeks of stroke.

Ten Sande et al. [31] reported the use of allogenic and xenogeneic stem cells as a potential alternative to autologous cells in the treatment of myocardial infarction, investigating the differences in terms of proarrhythmic effects of adipose-derived stromal cells (ADSCs) across species. Using microelectrode arrays and microelectrode recordings, the authors obtained local unipolar electrograms and action potentials from monolayers of neonatal rat ventricular myocytes (NRVMs) that were cocultured with rat, human, or pig ADSCs (rADSCs, hADSCs, or pADSCs, respectively). Monolayers of NRVMs were cultured in the respective conditioned medium to investigate paracrine effects, observing significant conduction slowing in all cardiomyocyte cultures containing ADSCs, independent of species used (*p* < 0.01).

Todorov et al. [32] tested the hypothesis that freshly harvested adipose-derived SVFs enhance devitalized hypertrophic cartilage (HC) remodeling into bone tissue. This investigation validates an innovative bone substitute material based on allogenic HC that is engineered, devitalized, stored, and clinically used, together with autologous cells, intraoperatively derived from a lipoaspirate.

Lee et al. [33] showed the safety and efficacy of allogenic AD-MSCs in treating lateral epicondylosis (LE). In this investigation, allogenic AD-MSCs combined with fibrin glue were infiltered into the hypoechoic common extensor tendon lesions of twelve patients with chronic LE; six patients each were administered 10^6^ or 10^7^ cells in 1 mL. Efficacy was analyzed by visual analog scale (VAS) score for elbow pain, modified Mayo clinic performance index for the elbow, and by evaluating ultrasound images of tendon lesions after 6, 12, 26, and 52 weeks. From baseline through 52 weeks of periodic follow-up, VAS scores progressively decreased, elbow performance scores improved, and tendon lesions also significantly decreased, confirming that allogenic AD-MSCs were safe and effective.

Zheng et al. [34] described the treatment of ARDS with allogenic AD-MSCs in a randomized, placebo-controlled pilot study. There were no serious side effects related to MSCs administration, and there were no significant differences in the overall number of adverse events between the two groups. In the MSCs group, serum Surfactant Protein-D (SP-D) levels at day 5 were significantly lower than those at day 0 (*p* = 0.027), while the changes in IL-8 levels were not significant. The IL-6 levels at day 5 showed a trend towards lower levels as compared with day 0, concluding that the administration of allogenic AD-MSCs appears to be safe and feasible in the treatment of ARDS.

Rozier et al. [35] reviewed clinical data reporting the allogenic use of MSCs derived from adipose tissue in systemic sclerosis.

Porzionato et al. [36] described, in a very interesting study, many decellularization protocols of ECM regarding the adipose tissue and other sources.

Perlee et al. [37] tested the administration of allogenic MSCs in sepsis, determining their effect on the response to intravenous lipopolysaccharide (LPS) in a randomized study in thirty-two healthy patients with four treatment arms: placebo or allogenic ASCs intravenously at either 0.25 × 10^6^, 1 × 10^6^, or 4 × 10^6^ cells/kg; all patients received LPS intravenously (2 ng/kg) one hour after the end of ASCs infusion. The infusion of ASCs was well tolerated. The high ASC dose increased the febrile response, exerted mixed pro-inflammatory (enhanced IL-8 release) and anti-inflammatory effects (increased IL-10 and TGF-β release), enhanced coagulation activation, and reduced the fibrinolytic response. These outcomes indicate that intravenous administration of allogenic ASCs (4 × 10^6^ cells/kg) has a variety of pro-inflammatory, anti-inflammatory, and procoagulant effects during human endotoxemia.

### 2.3. Critrical Assessment of Study Design

Performing a deep analysis of the selected studies during this investigation, it was possible to highlight its lack of a standardized and widely shared protocol for the decellularization procedures of AD and ECM, as well as standardized evaluation procedures. In particular, there is a lack of widely shared consensus on the decellularization procedure, on the recellularization method, and on the allogenic use of ASCs and SVFs. Additionally, the difficulty in clearly interpreting results was determined by the widely range of the studies analyzed (from pilot studies to randomized trials).

### 2.4. Side Effects

No major side effects have been displayed in the analyzed papers. Only tolerable and temporary pain during the procedures and transient edema have been described by some patients after allogenic ASCs infusion.

## 3. Discussion

It is fundamental that the implementation of adipose-derived stem cell therapy is based on the standard principles of EBM. Currently, cell therapy is considered as being equivalent to pharmaceutical drugs, as explained in the institutional guidelines and international rules section.

This systematic review included 19 articles exclusively on human allogenic ASCs transplants also reporting that the decellularization of adipose tissue and ECM appears to be the critical point of this procedure.

No severe adverse events have been reported, but for only a small number of complications that can be related directly to the AD gathering, trauma directly produced by the injection, or the nature of the underlying condition being treated.

At the same time, it appears necessary for future studies systematically administering allogenic ASCs to be cautious so as to monitor for possible serious side effects.

Among the investigations analyzed using allogenic ASCs treatment, there was no clear evidence of clinical immune response. For the investigations testing the presence and later production of donor-specific antibodies, 19–34% of patients produced these antibodies, suggesting that there is, however, a cellular response occurring toward the allogenic cells [20,24].

The consequence of this still remains unknown. For this reason, it can be discussed whether the use of allogenic ASCs really offers a superior amount of advantages or disadvantages over autologous ASCs, as many of the treated conditions are not acute and life-threatening. On the basis of this last concept, it seems, simpler and safer to use autologous ASCs. Certainly, it appears to be essential to consider the pathology kind to treat, the alternative therapies availability with documented safety and efficacy outcomes, and, finally, the ASCs’ availability. The use of one’s own ASCs offers the great advantage of being completely autologous, requiring several times only “minimal manipulation” based prevalently on centrifugation, filtrations, separation, and cutting. Enzymatic digestion of fat tissue, which offers a better quantity of ASCs compared with centrifugation and filtration procedures, can be performed, in the full respect of the European rules and institutional guidelines, when the autologous cells are used for the same essential function and when they are transplanted in the same anatomical structure (fat-fat), maintaining the same characteristics. All these procedures, represented by fat gathering and processing leading to ASCs isolation, can be performed in one-step surgery by physicians with consolidated clinical practices testified by scientific publications in this field. One of the most significant advantages offered by the ASCs is represented by the great availability of the fat tissue (ASCs source) in the human body, in addition to the simplicity of the gathering and processing procedure. Using ASCs cell expansion and culture via “extensive manipulation” (only in GMP facilities) procedures could permit obtaining an almost unlimited number of cells and seems to be also a more realistic option if one was to consider fat-cellular bio-banking, either as autologous or allogenic treatment modalities. Already, some studies in this sense have been published with funding from companies seeking to offer off-the-shelf allogenic ASCs therapy [20,24].

During the last 10 years, there has been a great increase in the number of allogenic adipose-derived cell therapy randomized clinical trials, as analyzed here. For this reason, it is important to continue well-conducted randomized controlled trials with adequate blinding, which also includes the safety assessment, and, at the same time, it is important to perform systematic review and meta-analyses of these as a study of EBM 1.

This systematic review was favored by a—not so great—number of investigations that did clearly describe their method of assessing safety, the absence of adverse events, and results. On the other hand, an initial limitation of the present systematic review was the unclear description of some in vitro and in vivo studies reporting on experimental preclinical models.

Recently, interesting articles have appeared with the aim to promote human tissue regeneration in different fields, using a new kind of MSCs, represented by the human periodontal ligament stem cells in bone regeneration [38,39], widening the horizons of autologous and allogenic regeneration to hitherto unexplored fields.

In each case, without a standardized procedure, regenerative allogenic therapies would be difficult, if not simply unreliable.

## 4. Methods

### 4.1. Rules and Institutional Guidelines on How to Handle Adipose Tissue and Stromal Vascular Fraction

The fat tissue preparation procedures are regulated by the Food and Drug Administration (FDA) and European Medicines Agency (EMA).

The regulations are linked to related laws, which describe a complex authorization path for the execution of these procedures. Reference is made to Regulation No. 1394/2007 of the European Parliament for Advanced Therapies (http://www.trovanorme.salute.gov.it/norme/renderNormsanPdf?anno=0&codLeg=47245&parte=1%20&serie=), which introduces the definition of “tissue engineering products” (TEP). Cells and tissues should only be considered TEP if they undergo “significant manipulation”. The same regulation defines the difference between minimal and extensive manipulation.

By “extensive manipulation” of tissues and/or cells, we mean all those manipulations that can determine to stimulation of cell proliferation and/or cell activation. In particular, tissues and/or cells are considered ”tissue-engineered“ if they meet at least one of the following parameters (art. 2, 1394/2007):

1. Tissues and/or cells that have undergone significant handling;
2. Tissues and/or cells that have not undergone the manipulations (minimal) listed in Annex I (centrifugation, filtration, cutting, separation, shaping, grinding, immersion in antimicrobial suspension, immersion in antibiotic, sterilization, vitrification irradiation, cell purification, cell concentration, freeze-drying, freezing, cryopreservation);
3. Tissues and/or cells not intended to be used for the same/the same essential functions in the recipient and donor.

(Extract from Regulation No. 1394/2007 of the European Paliament (EC) and of the Council of November 13, 2007, on advanced therapy medicinal products amending Directive 2001/83/EC and Regulation (EC) No. 726/2004 (relevant text for European Environment Agency (EEA) purposes).

Currently, “extensive manipulations” can only be carried out in authorized structures defined as “Good Manufacturing Practices” (GMP) laboratories by limiting the clinical applications of stem cells to centers equipped with these structures, whether they are of hematopoietic nature or of SVF. In fact, in centers without these facilities, these procedures cannot be performed by limiting the activity to the “minimum manipulations”, which can only be carried out in facilities equipped with personnel with consolidated clinical practice, certified by titles, such as scientific publications, which demonstrate effective experience in this area.

“Minimum manipulations” not considered as TEP are centrifugation, filtration, cutting, separation, shaping, grinding, immersion in antimicrobial suspension, immersion in antibiotic, sterilization, vitrification irradiation, cell purification, cell concentration, freeze-drying, freezing, and cryopreservation.

To complete the description of the rules available in the European legislative panorama and the translation of extracts related to the subject matter, the authors note the “Reflection paper on classification of advanced therapy medicinal products” of May 21, 2015, EMA/CAT/600280/2010 rev.1 Committee for Advanced Therapies (CAT) (http://www.ema.europa.eu/docs/en_GB/document_library/Scientific_guideline/2015/06/WC500187744.pdf).

Briefly, here were reported the Criteria for Somatic Cell Therapy Medicines (sCTMP) and TEP:

1. sCTMP and TEP contain or consist of engineered tissues and/or cells. To be considered “engineered”, tissues or cells must meet at least one of the following criteria:
  - Extensive manipulation (cell culture based on expansion of cells, genetic modification of these, their differentiation/stimulation with Growth Factors (GFs);
  - Various essential functions (not homologous use).

Cells or tissues not extensively or substantially manipulated, employed for the same essential function, must not be considered advanced therapy medicinal products (ATMPs). The same essential function means that cells gathered from their original anatomical microenvironment are used to maintain original capabilities and functions in the same anatomical or histological microenvironment. Therefore, not substantially modified cells, coming from various sources, for example, the fat cells (ASCs, SVFs) transplanted in a way/site different from the adipose tissue, are considered Advanced Therapy Medicinal Products (ATMPs). ATMPs may be considered all the cells that underwent a significant manipulation and/or for allogenic use.

All the procedures aimed to obtain ATMPs must be performed in agreement with the FDA regulations and European rules, institutional guidelines, and they must be realized following the principles outlined in the Declaration of Helsinki and internationally consented ethics in clinical research [40], performing a quality assessment based on the Strengthening the Reporting of Observational Studies in Epidemiology (STROBE) checklist [41], in addition to respecting the local laws. 

For all researchers, physicians, and, in particular, plastic surgeons, that want to use ASCs and SVFs in this field, the authors suggest strictly observing the guidelines highlighted in the European rules, as it has been reported to follow all the Good Clinical Practices (GCPs). 

The present investigation has been developed in agreement with research contract #1467/2017 between the first investigator, P.G., and the University “Tor Vergata”, Rome, Italy.

### 4.2. Search Strategy

This systematic review was registered in International prospective register of systematic reviews (PROSPERO, https://www.crd.york.ac.uk/prospero/#recordDetails) with ID code number: CRD42020160009.

**Figure 1:**
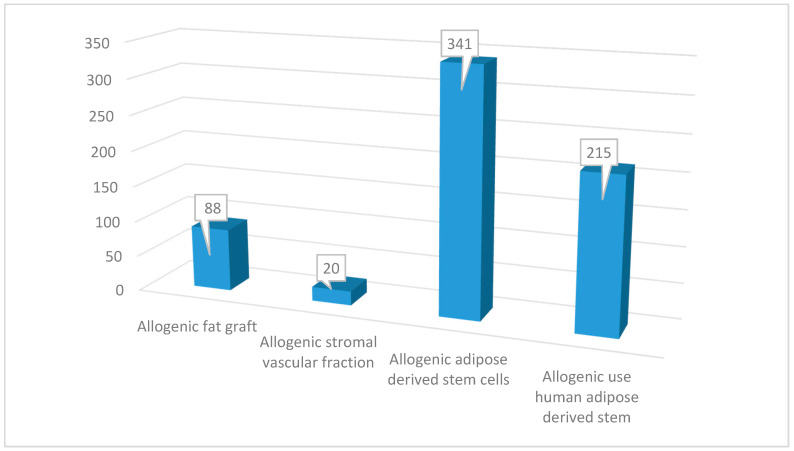
Papers initially found on allogenic adipose tissue applications.

The study was realized in agreement with the statements and guidelines of the Preferred Reporting Items of Systematic Reviews and Meta-analyses (PRISMA) [42] and the Cochrane handbook. A multistep research of the PubMed, MEDLINE, PreMEDLINE, Embase, PsycINFO, CINAHL, Clinicaltrials.gov, Cochrane, and Scopus databases has been conducted using different keywords (“allogenic fat graft”, “allogenic stromal vascular fraction”, “allogenic adipose-derived stem cells”, “allogenic use human adipose derived-stem cells”). In total, 664 articles were initially found on allogenic fat graft (*n* = 88), allogenic stromal vascular fraction (*n* = 20), allogenic adipose-derived stem cells (*n* = 341), allogenic use human adipose-derived stem (*n* = 215), as reported in Figure 1.

#### 4.2.1. Study Assessment

The objective of this investigation was to assess the selected articles based strictly on the human allogenic ASCs and SVFs transplants for clinical use. Articles included in this investigation had to match predetermined criteria in accordance with the PICOS (P—Patients, I—Intervention, C—Comparator, O—Outcomes, S—Study Design) approach. Inclusion and exclusion criteria are specified as following: P-Patients (inclusion criteria: age 18–80 years, males and females who suffered from each type pathologies; exclusion criteria: use of additional pharmacological therapeutics, bone marrow aplasia, uncompensated diabetes, sepsis, cancer; I-Intervention (inclusion criteria: human allogenic fat transplants, human allogenic ASCs and/or SVFs transplants, human allogenic ECM decellularized; exclusion criteria: human autologous fat transplant, human autologous ASCs and/or SVFs transplant, human autologous ECM decellularized, pre-clinical investigations, animal studies not related to human allogenic transplants, in vitro studies); C—Comparator (inclusion criteria: any control kind, internal, external, and different product; exclusion criteria: not performed); O—Outcomes (inclusion criteria: clinical evaluation, instrumental analysis, in vitro evaluation on human allogenic transplants; exclusion criteria: not performed); *S*—Study Design (inclusion criteria: randomized placebo-controlled trial/randomized, blinded, double-blind, clinical trials, review, systematic review, metanalyses; exclusion criteria: letter to editor, comments, expert opinion, case report, articles identified as bias (not correct match with the keyword used, group of study <10 patients, shorter follow-up than 3 months). No restrictions were applied to ethnicity or method of ASCs/SVFs/Fat processing.

This systemic review, conducted following the PICOS approach is considered an Evidence-Based Medicine (EBM) 1A level investigation according to the Oxford Centre for Evidence-Based Medicine (OCEBM), March 2009 (https://www.cebm.net/2009/06/oxford-centreevidence-based-medicine-levels-evidence-march-2009/).

#### 4.2.2. Study Selection

**Figure 2:**
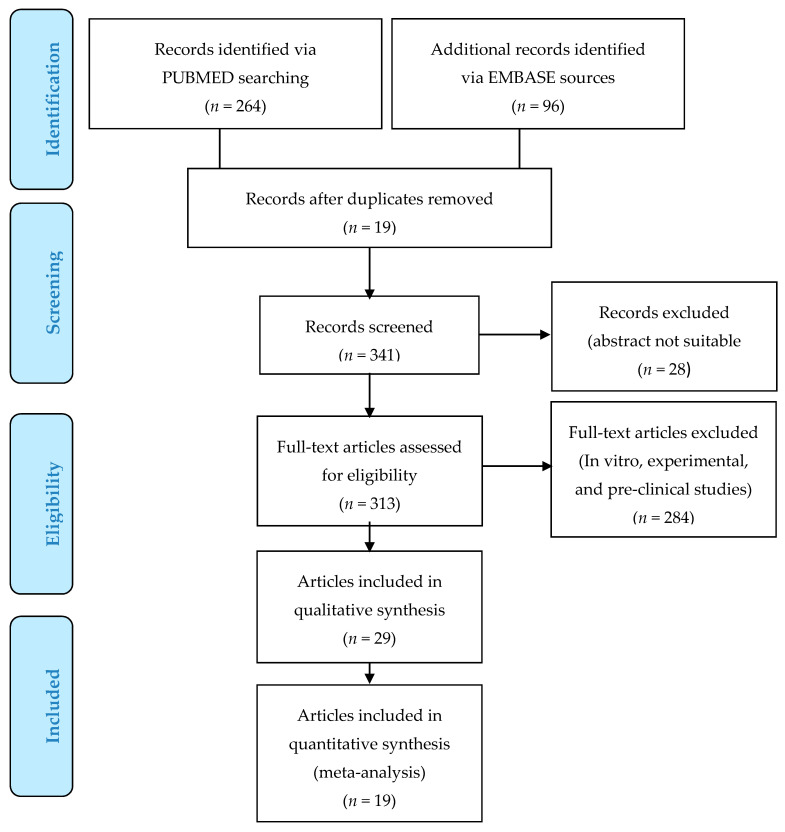
PRISMA Flow (Preferred reporting items for systematic review and meta-analysis). Articles selected (allogenic use of adipose tissue and adipose stem cells (ASCs) for clinical implications).

Three hundred and sixty articles focused on allogenic use of AD were initially identified using Prisma Flow (http://www.prisma-statement.org). Three hundred and forty-one published articles were reviewed after the exclusion of 19 duplicates. Only articles on AD human allogenic use were considered, and, for this reason, a total of 284 articles (in vitro, experimental, and pre-clinical studies) were excluded. The authors decided to select the only articles reporting the allogenic use of human ASCs for in vivo application. Duplicate studies, in vitro, and animal articles were discarded, screening only relevant papers according to title and abstracts. No time or language limits were adopted. Only 29 articles, cited in the text, were analyzed and considered using PRISMA flow (Figure 2).

Articles focused on complex perianal fistulas, diabetic foot ulcers, knee osteoarthritis, ARDS, refractory rheumatoid arthritis, pediatrics disease, fecal incontinence, allogenic flap, ischemic heart disease, autoimmune encephalomyelitis, lateral epicondylitis, soft tissue defects, and COVID-19 were identified and selected using Prisma Flow. Consequently, it will be decided to include only clinical trials with male and female patients diagnosed with these pathologies.

#### 4.2.3. Data Extraction

Data were independently gathered by the first author (P.G.) and checked by the last author (S.G.). Disagreement on gathered data was settled by consensus between P.G. and S.G. Several parameters were considered in data extraction, like first author, publication year, study design, patient number, procedures kind, and primary and secondary measures. The quality of the included articles and investigations was independently assessed by two researchers (P.G. and S.G.) using the Cochrane Collaboration’s Risk of Bias Assessment tool for randomized clinical trials and using the Newcastle–Ottawa Scale (http://www.ohri.ca/programs/clinical_epidemiology/oxford.asp) to evaluate the individual non-randomized studies.

#### 4.2.4. Outcome Measures

The primary outcomes were the safety and efficacy of allogenic human ASCs transplants for clinical use. Secondary outcomes were side effects. 

All outcomes gathered from the studies were reported with the same measurements retrieved from the papers. The placebo was used as a control in some of the included investigations, while, in other papers, patients were, respectively, allocated into study groups when they underwent allogenic ASCs transplant and to the control group when they underwent the placebo or other treatments.

## 5. Conclusions

The human allogenic use of ASCs appears to be safe and effective in the studies analyzed. No severe adverse effects were found. In each case, while autologous ASCs therapy has shown great clinical application in many fields, it appears to be necessary to perform additionally higher-quality studies before considering it as a standard cellular therapy the allogenic use of ASCs.

The investigators believe that the next future will be focused prevalently on autologous- and allogenic regenerative-based therapies and, for this reason, invite all the audience to improve the standard quality of articles in this field publishing prevalently EBM level 1 articles.

## Abbreviations

SVFs	Stromal Vascular Fraction Cells
ASCs	Adipose-Derived Stem Cells
SVF	Stromal Vascular Fraction
ISCT	International Society for Cellular Therapy
MSCs	Mesenchymal Stem Cells
HAT	Human Adipose Tissue
COVID-19	Coronavirus Disease 2019
UC	Umbilical-Cord
BM	Bone-Marrow
BM-MSCs	Bone-Marrow MSCs
UC-MSCs	Umbilical-Cord MSCs
AD	Adipose Tissue
AD-MSCs	Adipose-Derived Mesenchymal Stem Cells
ECM	Extracellular Matrix
FDA	Food and Drug Administration
EMA	European Medical Agency
CAT	Committee for Advanced Therapies
EC	European Parliament
GMP	Good Manufacturing Practices
GCP	Good Clinical Practices
sCTMP	Somatic Cell Therapy Medicines
TEP	Tissue Engineering products
ATMPs	Advanced Therapy Medicinal Products
STROBE	Strengthening the Reporting of Observational Studies in Epidemiology
PRISMA	Preferred Reporting for Items for Systematic Reviews and Meta-Analyses
EBM	Evidence-Based Medicine
OCEBM	Oxford Centre for Evidence-Based Medicine
ARDS	Acute Respiratory Distress Syndrome
haMPCs	allogenic human Adipose-Derived Mesenchymal Progenitor Cells
e-ASC	expanded Adipose-derived Stem Cells
RA	Rheumatoid Arthritis
OA	Osteoarthritic
e-a-MSCs	expanded-allogenic Mesenchymal Stem Cells
CSCC_ASC	Cardiology Stem Cell Centre Adipose-derived Stromal Cell
IHF	Ischemic Heart Failure
GvHD	Graft-versus-Host disease
ADSCs	Adipose-derived Stromal Cells
NRVMs	Neonatal Rat Ventricular Myocytes
HC	Hypertrophic cartilage
LE	Lateral Epicondylosis
VAS	Visual Analog Scale
LPS	Lipopolysaccharide
